# Evidence of natural Zika virus infection in neotropical non-human primates in Brazil

**DOI:** 10.1038/s41598-018-34423-6

**Published:** 2018-10-30

**Authors:** Ana Carolina B. Terzian, Nathalia Zini, Lívia Sacchetto, Rebeca Froes Rocha, Maisa Carla Pereira Parra, Juliana Lemos Del Sarto, Ana Carolina Fialho Dias, Felipe Coutinho, Jéssica Rayra, Rafael Alves da Silva, Vivian Vasconcelos Costa, Natália Coelho Couto De Azevedo Fernandes, Rodrigo Réssio, Josué Díaz-Delgado, Juliana Guerra, Mariana S. Cunha, José Luiz Catão-Dias, Cintia Bittar, Andréia Francesli Negri Reis, Izalco Nuremberg Penha dos Santos, Andréia Cristina Marascalchi Ferreira, Lilian Elisa Arão Antônio Cruz, Paula Rahal, Leila Ullmann, Camila Malossi, João Pessoa de Araújo Jr, Steven Widen, Izabela Maurício de Rezende, Érica Mello, Carolina Colombelli Pacca, Erna Geessien Kroon, Giliane Trindade, Betânia Drumond, Francisco Chiaravalloti-Neto, Nikos Vasilakis, Mauro M. Teixeira, Maurício Lacerda Nogueira

**Affiliations:** 1 São José do Rio Preto School of Medicine (FAMERP), Avenida Brigadeiro Faria Lima, 5416, CEP: 15090-000, Vila São Pedro, São José do Rio Preto, SP Brazil; 2 Laboratório de Vírus - Universidade Federal de Minas Gerais (UFMG), Avenida Antônio Carlos, 6627, CEP: 31270-901, Pampulha, Belo Horizonte, MG Brazil; 3 Center for Drug Research and Development, Instituto de Ciências Biológicas, Universidade Federal de Minas Gerais (UFMG), Avenida Antônio Carlos, 6627, CEP: 31270-901, Pampulha, Belo Horizonte, MG Brazil; 4 Instituto Adolfo Lutz (IAL), Avenida Dr. Arnaldo, 351 − 7 Andar, Sala 706, CEP: 01246-000, Pacaembú, São Paulo, SP Brazil; 5 Laboratory of Wildlife Comparative Pathology, Department of Pathology, School of Veterinary Medicine and Animal Sciences, University of São Paulo (LAPOCM-FMVZ-USP), Avenida Orlando Marques de Paiva, 87, CEP: 05508-270, São Paulo, SP Brazil; 6 Department of Biology, Institute of Biosciences, Letters, and Exact Sciences – São Paulo State University, São José do Rio Preto – (IBILCE/UNESP), Rua Cristóvão Colombo, 2265, CEP: 15054-000, São José do Rio Preto, SP Brazil; 7 Epidemiological Surveillance Departament of São José do Rio Preto, Avenida Romeu Strazzi, 199, CEP: 15084-010, Vila Sinibaldi, São José do Rio Preto, SP Brazil; 8 São Paulo State University (Unesp), Institute for Biotechnology, Alameda das Tecomarias, s/n, CEP: 18607-440, Chácara Capão Bonito, Botucatu, SP Brazil; 9 Department of Biochemistry and Molecular Biology, University of Texas Medical Branch, 301 University Blvd, Galveston, TX 77555-0645 USA; 10 Centro de Controle de Zoonoses, Belo Horizonte Council, Rua Édna Quintel, 173, CEP: 31270-705, São Bernardo, Belo Horizonte, MG Brazil; 11 Faceres Medical School, Avenida Anísio Haddad, 6751, CEP: 15090-305, Jardim Francisco Fernandes, São José do Rio Preto, SP, Brazil; 12 Department of Epidemiology, School of Public Health of the University of São Paulo, Avenida Dr. Arnaldo, 715, CEP: 01246-904, São Paulo, SP Brazil; 13 Department of Pathology and Center of Biodefense and Emerging Infectious Diseases, Center for Tropical Diseases, Institute for Human Infections and Immunity, University of Texas Medical Branch, 301 University Blvd, Galveston, TX 77555-0609 USA

## Abstract

In Africa, Old World Primates are involved in the maintenance of sylvatic circulation of ZIKV. However, in Brazil, the hosts for the sylvatic cycle remain unknown. We hypothesized that free-living NHPs might play a role in urban/periurban ZIKV dynamics, thus we undertook an NHP ZIKV investigation in two cities in Brazil. We identified ZIKV-positive NHPs and sequences obtained were phylogenetically related to the American lineage of ZIKV. Additionally, we inoculated four *C*. *penicillata* with ZIKV and our results demonstrated that marmosets had a sustained viremia. The natural and experimental infection of NHPs with ZIKV, support the hypothesis that NHPs may be a vertebrate host in the maintainance of ZIKV transmission/circulation in urban tropical settings. Further studies are needed to understand the role they may play in maintaining the urban cycle of the ZIKV and how they may be a conduit in establishing an enzootic transmission cycle in tropical Latin America.

## Introduction

Zika virus (ZIKV) is a emerging flavivirus similar to dengue (DENV), yellow fever (YFV) and Chikungunya (CHIKV) viruses that share the same vector *Ae*. *aegypti*, in the urban transmission cycle^1^. However, unique to ZIKV there are a number of non-vectored modes of transmission that may involve blood transfusion, sexual intercourse, and *in utero* or *ex utero* maternal transmission^2^. Humans are considered the only reservoir host in the ZIKV urban/peri-urban transmission cycle, while several species of NHPs have been implicated in the sylvatic transmission cycle^3^ [reviewed in^4^]. Indeed, the first ZIKV isolation was in Uganda in 1947, in a sentinel rhesus monkey (*Macaca mulatta*), during a yellow fever virus serosurvey^5^. Several epizootics were reported in several Old world primates (OWP) species at the time likely involving ZIKV^6,7^.

A critical factor for the development of public health strategies to mitigate the risk of continued human ZIKV disease is whether a sylvatic cycle of ZIKV transmission is likely to become established in South America, as occurred centuries ago for yellow fever virus after its importation from Africa. There are several factors that may contribute to the potential for enzootic transmission of ZIKV (e.g., susceptible vectors, vector density, proximity to humans with ZIKV infection and environmental factors) and given the broad diversity of Neotropical NHPs in South America, the large numbers of primates living in the Amazon Basin and the proximity of some species (e.g., *Saimiri*, *C*. *penicillata*) to human activities, understanding the susceptibility of Neotropical NHPs to ZIKV infection is critical for predicting future ZIKV circulation.

There is increasing evidence to suggest that New World Primates (NWP or Neotropical NHPs) may be susceptible to ZIKV infection, since ZIKV RNA was detected in four free-living common marmosets and three black-striped capuchin (*Sapajus libidinosus*)^8^. The common marmoset (*Callithrix jacchus*) is a small NWP native to eastern Brazil, which diverged from the OWP approximately 26 to 27 million years ago^9^. *Callithrix penicillata* (C. penicillata) or black-tuffed marmosets are a native species of Cerrado, a vast ecoregion in Brazil that covers up to 20% of the territory. *C*. *penicillata* is one of the most adaptable species of the *Callithrix* genus giving its ability to explore inhospitable regions and exploit tree exudates. Therefore, as consequence of anthropological interference, this species was introduced in urban territories, especially in southern and southeastern metropoleis that houses half of the total population of Brazil. Importantly, *C*. *penicillata* is present in urban areas highly infested with *Aedes sp* mosquitoes^10^.

A recent study experimentally examined whether two widespread species of Neotropical NHPs, squirrel monkeys (*Saimiri* spp.) and owl monkeys (*Aotus* spp.) can serve as a reservoir and amplification host for ZIKV^11^. For both species viremia in the absence of detectible disease was observed and seroconversion occurred by day 28. ZIKV was also detected in the spleen of three owl monkeys. While this seminal study confirmed the susceptibility to ZIKV infection of NHPs that live in close proximity to humans, it raised the possibility that establishment of a ZIKV sylvatic transmission cycle in South America, which in turn could render eradication efforts impossible to implement thus provide a mechanism for continued exposure of humans to ZIKV infection and disease.

Common marmosets (family *Callitrichidae*), have been extensively studied as an experimental pathogenesis model for a number of tropical arboviral diseases^12^. Recently, a ZIKV study using male and pregnant common marmosets demonstrated that the animals recapitulated the characteristics of ZIKV infection in humans. While adult males did not present and signs of clinical disease, viremia was detected resulting in their seroconversion and protection from subsequent ZIKV infection^13^. Likewise, the pregnant marmoset dams exhibited prolonged viremia, viruria and seroconversion, as previously observed in humans^14,15^, as well as viral replication in the placenta^16^.

In this study, we hypothesized that free-living NHP might play a role in urban/periurban ZIKV transmission. Initially, we investigated the possibility of ZIKV infection in tissues of NHP carcasses (*Callithrix* sp. and *Sapajus* sp.) collected in cities of São Paulo and Minas Gerais States, Brazil. Our results provided evidence of natural ZIKV infection among wild marmosets in São Paulo and Minas Gerais States. We then analyzed mosquitoes collected in the same region and time that the NHP carcasses were collected in São Paulo State, and we detected ZIKV positive mosquito pools. To investigate further these observations, we inoculated four *C*. *penicillata* with a low passage ZIKV isolate from a human patient. Viral serological and molecular analyses revealed that marmosets had a sustained viremia post inoculation, suggesting that these NHPs have the potential to serve as a reservoir and amplification host in the urban transmission cycle.

## Results

### Viral molecular investigation

From 82 NHP carcasses (free-living marmosets and capuchin) received and analyzed, from January to June of 2017, 32 (39.02%) were ZIKV-positive in at least one tissue sample. In these animals, the Ct (cycle threshold) values ranged from 27.64 to 38.47 (Table 1). The tissues that presented higher positivity were kidneys (n = 18; ct: 29.17 to 38.47), liver (n = 18; ct: 29.18 to 37.88), brain (n = 15; ct: 30.4 to 37.71), spleen (n = 13; ct: 30.68 to 38.37), lungs (n = 11; ct: 27.76 to 38.37), heart (n = 9; ct: 29.72 to 37.85), and gonads (n = 1; ct: 36.3). All the 82 NHP carcasses analyzed were negative for YFV.

**Table 1:** Non-human primates positive for Zika virus, by RT-qPCR.

Sample ID	NHPs species	Organs (Ct value)						
		KIDNEY	BRAIN	SPLEEN	LUNG	LIVER	HEART	GONADS
PR 17/02	*Callithrix* sp.	n/a	n/a	38.37	n/a	n/a	n/a	n/a
PR 17/03	*Callithrix* sp.	n/a	37.65	n/a	neg	neg	n/a	n/a
PR 17/04	*Callithrix* sp.	37.84	neg	38.37	38.37	neg	36.19	n/a
PR 17/05	*Callithrix* sp.	38.19	36	35.14	36.37	37.88	neg	n/a
PR 17/06	*Callithrix* sp.	37.96	neg	38.23	neg	neg	n/a	n/a
PR 17/07	*Callithrix* sp.	38.18	37.71	neg	37.92	neg	neg	n/a
PR 17/08	*Sapajus* sp.	35.98	34.44	38.91	neg	neg	neg	n/a
PR 17/11	*Callithrix* sp.	Neg	37.36	neg	neg	neg	neg	n/a
PR 17/12	*Callithrix* sp.	35.66	35.64	neg	n/a	37.27	neg	n/a
PR 17/13	*Callithrix* sp.	35.57	32.31	37.13	neg	29.25	neg	n/a
PR 17/14	*Callithrix* sp.	Neg	37.12	37	neg	neg	neg	n/a
PR 17/15	*Callithrix* sp.	Neg	neg	neg	neg	37.51	n/a	n/a
PR 17/16	*Callithrix* sp.	30.87	30.4	31.48	27.76	29.18	31.32	n/a
PR 17/17	*Callithrix* sp.	31.66	31.13	n/a	31.75	32.13	30.36	n/a
PR 17/18	*Callithrix* sp.	29.17	31.86	32.87	30.47	30.16	29.72	n/a
PR 17/19	*Callithrix* sp.	32.65	32.38	31.4	31.89	neg	32.32	n/a
PR 17/20	*Callithrix* sp.	34.68	31.11	neg	34.36	neg	34.41	n/a
PR 17/21	*Callithrix* sp.	32.89	31.11	33.27	31.9	32.86	32.59	n/a
PR 17/22	*Callithrix* sp.	29.11	31.86	30.68	34.06	31.56	34.47	n/a
PR 17/23	*Callithrix* sp.	Neg	neg	n/a	neg	neg	37.85	n/a
PR 17/25	*Callithrix* sp.	37.79	neg	34.75	37.28	36.44	neg	n/a
PR 17/26	*Callithrix* sp.	37.69	n/a	35.94	neg	neg	36.97	n/a
PR 17/27	*Callithrix* sp.	38.47	neg	neg	neg	neg	neg	n/a
MG 17/01	*Callithrix* sp.	n/a	n/a	n/a	n/a	36.0	n/a	n/a
MG 17/02	*Callithrix* sp.	n/a	n/a	n/a	n/a	33.9	n/a	n/a
MG17/15	*Callithrix* sp.	27.1	n/a	n/a	n/a	n/a	n/a	neg
MG 17/16	*Callithrix* sp.	n/a	n/a	n/a	n/a	35.3	n/a	n/a
MG17/30	*Callithrix* sp.	neg	n/a	n/a	n/a	35.6	n/a	36.3
MG 17/31	*Callithrix* sp.	n/a	n/a	n/a	n/a	35.9	n/a	n/a
MG 17/32	*Callithrix* sp.	n/a	n/a	n/a	n/a	36.3	n/a	n/a
MG 17/45	*Callithrix* sp.	n/a	n/a	n/a	n/a	35.6	n/a	n/a
MG 17/51	*Callithrix* sp.	n/a	n/a	n/a	n/a	35.7	n/a	n/a
**Mosquitoes (Ct Value)**								
**Sample ID**	**Mosquito species**	**Ct**						
17/151	*Ae*. *aegypti*	35.17						
17/160	*Ae*. *aegypti*	36.89						
17/161	*Ae*. *aegypti*	36.48						
17/163	*Ae*. *aegypti*	31.87						
17/164	*Ae*. *aegypti*	36.77						
17/169	*Ae*. *aegypti*	22.23						

Positive samples and mosquitoes are indicated by the Ct (cycle threshold) value. n/a: not available. neg: negative. Samples collected in São José do Rio Preto (SP), from January to March 2017 are identified by PR followed by year and sample ID. Samples collected in Minas Gerais, from January to June 2017, are identified by MG followed by year and sample ID. Mosquitoes collected in São José do Rio Preto (SP), in the first trimester of 2017 are identified by year and sample ID.

**Figure 1:**
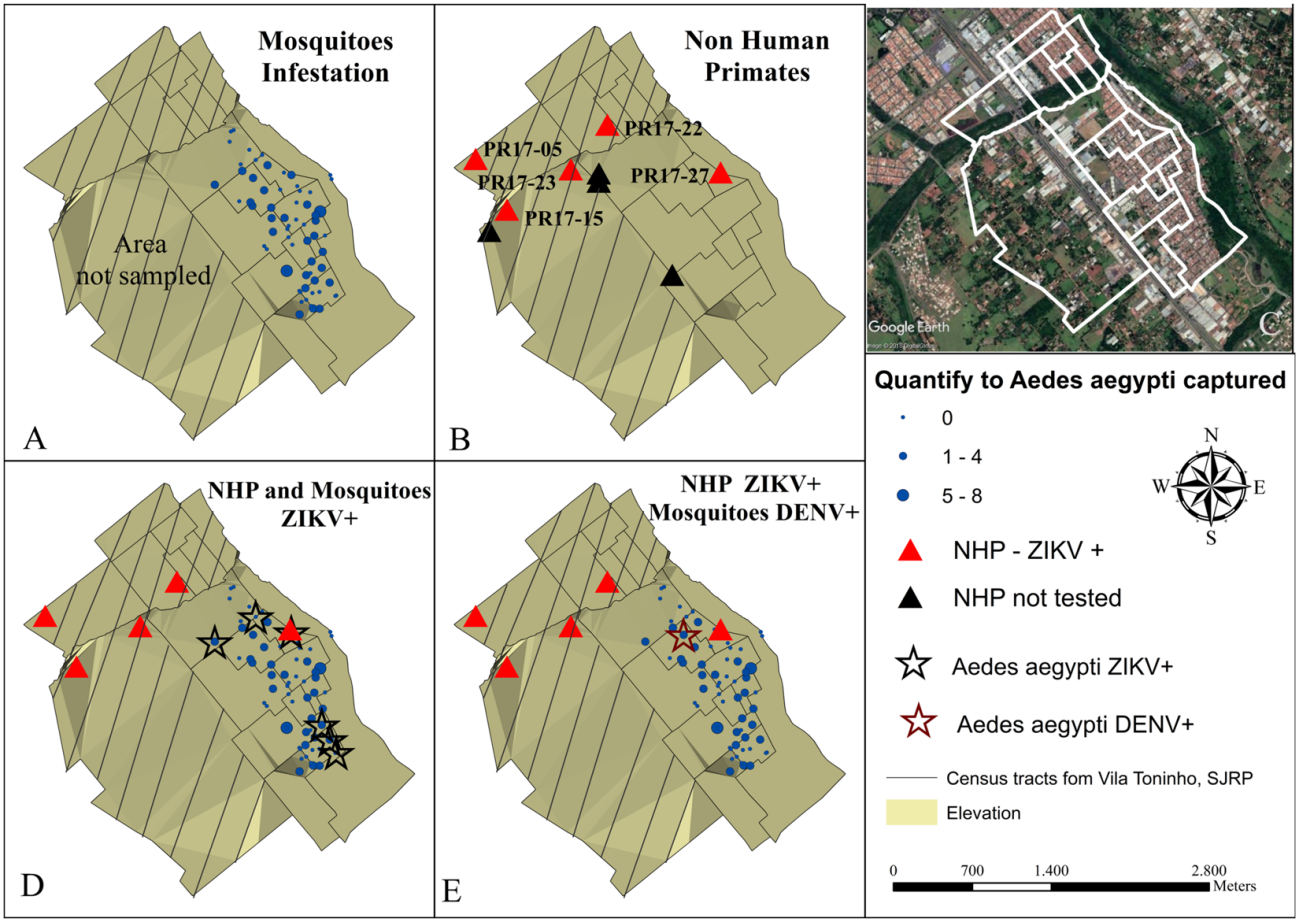
Geoprocessing map of the NHPs and mosquitoes captured in the Vila Toninho neighborhood. (**A**) Schematic representation of the area where mosquitoes are regularly collected in the Vila Toninho neighborhood. The hatched area represents the area where there is no specimen collection. The blue dots represent the collection points of the mosquitoes and the quantity of specimen collected. (**B**) Schematic representation of the collection points of the nine NHP found dead. The NHPs identified by ID PR 17-05, PR 17-15, PR 17–22, PR 17–23, PR 17–27 were analyzed and tested positive for ZIKV in one or more tissue samples and are represented by a red triangle. The black triangles represent the NHPs collected but not tested. (**C**) Satellite image of the Vila Toninho neighborhood. The boundary of the neighborhood is marked in white. Vegetation cover area can be seen in green surrounding the neighborhood. (**D**) Overlap of the area of the animals and mosquitoes collection. The ZIKV-positive PR 17–27 is overlapping with a ZIKV-positive *Ae*. *aegypti* mosquito pool. (**E**) Overlap of the areas of animas and mosquito collections with the presence of the DENV-positive *Ae*. *aegypti* mosquitoes **(**Vila Toninho satellite image by Google Earth Pro 7.3.1.4507 (64-bit) software. URL https://www.google.com/maps/@−20.84677,−49.34063,5682 m/data = !3m1!1e3). Map data: Google, 2018 DigitalGlobe.

During the first trimester of 2017, 74 *Ae*. *aegypti* and three *Ae*. *albopictus* mosquitoes were collected in Vila Toninho and divided into 27 pools (25 pools of *Ae*. *aegypti* and two pools of *Ae*. *albopictus*). Six pools were ZIKV-positive (Table 1) and one pool was DENV-positive (Fig. 1A–E). None of the mosquito pools was positive for YFV and none of the *Ae*. *albopictus* pools tested positive for any of the arboviruses. During the same period, the Bretou Index (BI) was performed in 649 residences of the neighborhood, where 35 were positive for *Ae*. *aegypti* presence (BI = 5.4) and five were positive for *Ae*. *albopictus* presence (BI = 0.8).

In order to confirm and characterize the ZIKV genome detected in NHPs, we attempted nucleotide sequencing. Initially, the total RNA extracted from NHP RT-qPCR-positive carcasses for ZIKV was evaluated for sequencing, however it was considered of low integrity and degraded and thus not suitable for library preparation, according to the manufacturer’s instructions. However, since these were the only available samples, we decided to not consider RNA integrity as an exclusion criterion for library preparation in this case, since these were the available samples, and we were able to prepare libraries from four NHP.

**Figure 2:**
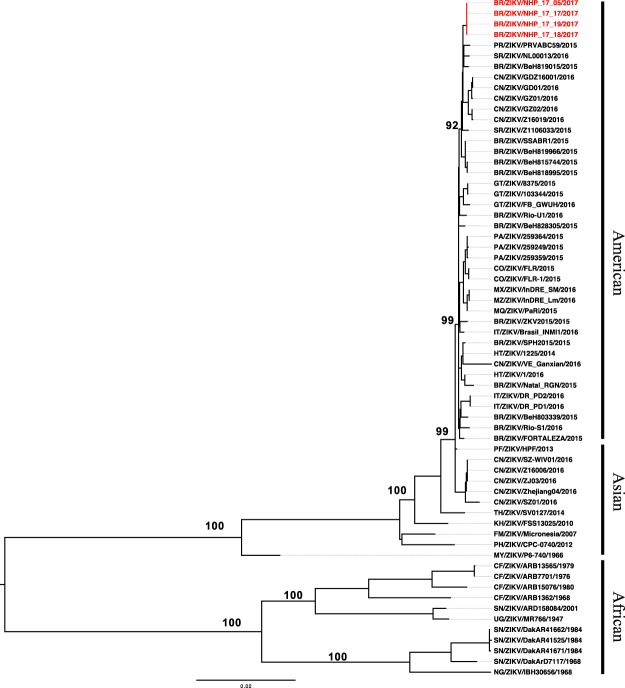
Molecular Phylogenetic analysis of Zika virus by the Maximum Likelihood method. The four strains obtained from NHPs (marmosets) are highlighted in red. Bootstrap values above 90% are shown. Initial tree(s) for the heuristic search were obtained automatically by applying Neighbor-Join and BioNJ algorithms to a matrix of pairwise distances estimated using the Maximum Composite Likelihood (MCL) approach, and then selecting the topology with superior log likelihood value. A discrete Gamma distribution was used to model evolutionary rate differences among sites (5 categories (+G, parameter = 1.7699)). The rate variation model allowed for some sites to be evolutionarily invariable ([+I], 52.6922% sites). The tree was drawn to scale, with branch lengths measured in the number of substitutions per site. There were a total of 10269 positions in the final dataset. Evolutionary analyses were conducted in MEGA7^64^.

DNA libraries within the 200–300 bp range were prepared and evaluated on the Agilent chip with a concentration of 5.57 nM on RT-qPCR quantification. Sequencing run resulted in 41,445,050 reads with 97.08% passing filter reads (38,870,718 reads) and 97.04% had quality ≥Q30. On Geneious R8 analysis using a ZIKV genome as a reference, a variable number of reads/sample mapped to the ZIKV genome, as follows: 515 reads from sample PR 17/05: 1,616 reads from sample PR 17/17; 356 reads from sample PR 17/18, and 1,446 reads from sample PR 17/19. The obtained sequences were used to perform phylogenetic inferences, using Maximum likelihood (Fig. 2) and Bayesian methods (Supplementary Fig. S1). Both analyses showed that the four ZIKV sequences originated from marmosets carcasses (PR17/05, PR 17/17, PR 17/18 and PR 17/19) clustered together, within the newly emerged American lineage (GenBank accession number: MG770183, MG770184, MG770185, MG770186).

### Histopathology and Immunohistochemistry Observations

A total of 16 ZIKV (RT-qPCR)-positive NHPs, from São José do Rio Preto had samples analyzed (Table 2). Fourteen of 16 (88%) NHP carcasses had interstitial pneumonia, bronchopneumonia, pulmonary edema and/or bronchiolitis. Thirteen of 16 (81%) NHP carcasses had cholangiohepatitis, cholangitis, hepatocellular single cell necrosis/apoptosis and/or diffuse hydropic hepatocellular degeneration. Eleven of 16 (69%) NHP carcasses had splenic lymphoid reactive hyperplasia, sinus histiocytosis, lymphoid depletion and/or necrotizing splenitis. Eleven of 16 (69%) NHP carcasses had interstitial nephritis, glomerulonephritis and/or tubular proteinosis. Seven of 16 (44%) NHP carcasses had brain hemorrhage, meningitis, encephalitis and/or acute cortical neuronal necrosis. Seven of 16 (44%) NHP carcasses had acute cardiomyocyte degeneration, hemorrhage and/or myocarditis. All animals were negative for YF virus antigen.

**Table 2:** Histopathologic findings in 16 NHPs positive for Zika virus.

Sample ID	Cerebrum	Heart	Lung	Liver	Spleen	Kidney
PR 17/03	Autolysis	Autolysis	Autolysis	Autolysis	Autolysis	Autolysis
PR 17/04	Autolysis	Autolysis	Mild multifocal suppurative bronchopneumonia; Intravascular microfilariae	Moderate multifocal chronic proliferative cholangitis with intralesional trematode ova and adults; Mild to moderate diffuse vacuolar (hydropic) hepatopathy; Intravascular microfilariae	Autolysis	Minimal focal lymphoplasmacytic interstitial nephritis
PR 17/05	Congestion and multifocal acute hemorrhage.	Congestion and multifocal acute hemorrhage.	Multifocal acute alveolar hemorrhage	Multifocal random mononuclear inflammatory infiltrates and minimal pericholangitis; Mild diffuse vacuolar (hydropic) hepatopathy	Mild to moderate, diffuse sinus histiocytosis	Minimal multifocal acute tubular degeneration with mild proteinosis; Minimal focal chronic lymphocytic interstitial nephritis
PR 17/07	NSLO	NSLO	Moderate to severe acute alveolar hemorrhage	Marked, diffuse vacuolar (hydropic) hepatopathy	Extramedullary hematopoiesis; Lymphoid reactive hyperplasia	Without NSLO
PR 17/08	NE	Minimal multifocal chronic lymphocytic myocarditis	Marked multifocal acute alveolar and bronchial hemorrhage with edema and histiocytosis; Mild multifocal chronic lymphocytic and eosinophilic perivasculitis and mild lymphoid hyperplasia; Focal parabronchial arterial bone marrow embolus.	Congestion; Minimal, focal, chronic eosinophilic pericholangitis and minimal reactive changes.	Diffuse lymphoid reactive hyperplasia and sinus histiocytosis	Mild, multifocal tubular proteinosis; Congestion.
PR 17/11	Multifocal acute cortical neuron necrosis	Congestion, edema and focal hemorrhage	Congestion, edema and focal hemorrhage	Moderate diffuse vacuolar (hydropic) hepatopathy	Diffuse lymphoid reactive hyperplasia	Moderate multifocal chronic membranous glomerulonephritis with lymphoid follicle formation
PR 17/12	NSLO	NSLO	Moderate multifocal acute alveolar hemorrhage and edema	Moderate diffuse vacuolar (hydropic) hepatopathy; Moderate multifocal neutrophilic portal hepatitis	Mild diffuse lymphoid reactive hyperplasia	Congestion
PR 17/13	Mild focal hemorrhage	Mild multifocal acute myocyte degeneration	Mild multifocal acute interstitial pneumonia	Marked multifocal chronic proliferative cholangiohepatitis with marked cholestasis and necrosis; Extramedullary hematopoiesis	Multifocal acute necrotizing splenitis; Extramedullary hematopoiesis.	NSLO
PR 17/14	Congestion	NSLO	Moderate multifocal subacute interstitial pneumonia with bronchiolitis	NSLO	Moderate lymphoid depletion	NSLO
PR 17/15	NE	NSLO	Autolysis	NSLO	NSLO	Mild multifocal chronic interstitial nephritis
PR 17/16	Congestion	Minimal focal chronic lymphocytic infiltrate; Congestion	Diffuse atelectasia; Mild multifocal subacute suppurative bronchopneumonia	Mild multifocal chronic lymphocytic pericholangitis; Mild diffuse vacuolar (hydropic) hepatopathy	NE	Mild multifocal chronic lymphocytic interstitial nephritis; Mild multifocal acute tubular degeneration
PR 17/18	Minimal focal lymphocytic vascular cuffing; Congestion and multifocal alveolar hemorrhage	Mild multifocal myocardial fibrosis; Mild multifocal acute myocyte degeneration with contraction band necrosis	Multifocal acute alveolar hemorrhage	Mild multifocal chronic eosinophilic and lymphoplasmacytic cholangitis with intraductal adult trematodes, bile duct hyperplasia and mild fibrosis	Mild diffuse lymphoid reactive hyperplasia and sinus histiocytosis	Mild multifocal chronic lymphoplasmacytic interstitial nephritis; Mild multifocal segmental membranous glomerulonephritis with proteinuria; Mild multifocal acute tubular necrosis
PR 17/19	Multifocal acute hemorrhage and congestion.	NSLO	Congestion, edema and multifocal acute alveolar hemorrhage; Focal interstitial nodular lymphohistiocytic and eosinophilic infiltrate	Mild multifocal chronic lymphoplasmacytic pericholangitis	Diffuse lymphoid reactive hyperplasia and histiocytosis	Moderate diffuse global chronic membranoproliferative glomerulitis with moderate lymphoplasmacytic interstitial nephritis; Focal granuloma.
PR 17/20	NE	NSLO	Locally extensive alveolar hemorrhage; Moderate focal acute suppurative bronchopneumonia	Mild diffuse vacuolar (hydropic) hepatopathy	NSLO	NSLO
PR 17/21	Mild focal chronic lymphocytic meningitis	Congestion	Diffuse congestion, edema and atelectasia; mild alveolar hemosiderosis	Mild multifocal chronic lymphoplasmacytic and granulomatous pericholangitis with mild hemosiderosis; Mild multifocal acute single cell hepatocellular necrosis	Mild diffuse lymphoid reactive hyperplasia and mild sinus histiocytosis	Mild multifocal acute tubular degeneration with proteinosis e atrofia glomerulocística focal
PR 17/22	NSLO	NSLO	Diffuse congestion, edema and multifocal acute hemorrhage; Multifocal atelectasia; Mild lymphoplasmacytic peribronchial and perivascular infiltrates with rare alveolar multinucleate giant cells	Mild multifocal chronic lymphoplasmacytic and eosinophilic cholangiohepatitis with moderate bile duct hyperplasia; Mild diffuse vacuolar (hydropic) hepatopathy	Moderate diffuse lymphoid reactive hyperplasia and sinus histiocytosis	Minimal multifocal chronic lymphoplasmacytic interstitial nephritis and rare glomerulocystic atrophy

NSLO: no significant lesions observed; NE: not evaluated.

### Geoprocessing

After the geoprocessing analyses, a cluster between mosquitoes and NHP positive for ZIKV was observed (Fig. 1D). As expected, we noticed the occurrence of DENV-positive mosquitoes in the same area at the same time. Due to the animals’ carcass conditions, which were dead upon collection, only five of the 9 animals had tissue samples that were amenable to study (PR 17/05, PR 17/15, PR 17/22, PR17/23, PR 17/27) (Tables 1 and 2). Among those five ZIKV-positive NHPs, the marmoset PR 17/27 overlapped with a ZIKV-positive *Ae*. *aegipty* pool. This data could suggest that the co-occurrence of ZIKV in *Ae*. *aegypti* and NHPs suggested the possible transmission between these urban vectors and primates, which could contribute to transmission in the area.

## Experimental Infection Results

### Viremia in infected *Callithrix penicilata*

**Figure 3:**
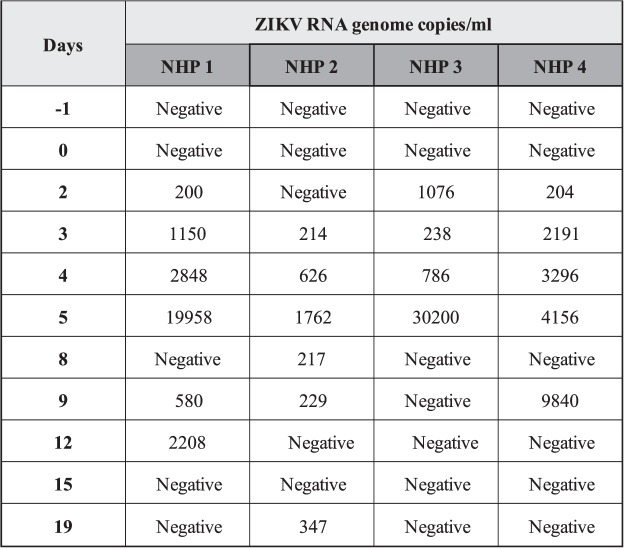
Viremia measurement in experimentally ZIKV-infected *Callithrix penicilata* collected from day −1 until 19 dpi. One-step qRT-PCR was used to measure semi quantitatively the ZIKV RNA loads in the serum of four animals at indicated days p.i. and represented as viral RNA copies per mL of sample standard curve. The curve was obtained from a standard sample with known titer after serial dilutions (5 × 10^1^ to 5 × 10^6^ copies/mL) on the plasma of the non-infected marmosets.Values are expressed by RNA genome copies per mL for all the infected marmosets. Viremia was detected in the serum of marmosets 1, 3 and 4 on day 2 p.i. and in all infected marmosets on day 3 p.i. The figure shows that viremia increased on day 5 p.i. when compared to other evaluated days for all the infected marmosets. p.i.: post infection. NHP: non-human primates. Day −1: day before the infection.

To evaluate viremia, four marmosets were infected with 10^5^ or 10^6^ PFU/mL of ZIKV (strain HS-2015-BA-01). Serum samples were collected on the day before infection (day −1) and on days 2, 3, 4, 5, 8, 9, 12, 15 and 19 p.i. Viremia was detected in the serum of marmosets 1, 3 and 4 on day 2 p.i. and in all infected marmosets on day 3 p.i. On the 5^th^ day p.i., viremia was increased in all four marmosets when compared with previews days, reaching a mean value of 1.4 × 10^4^ viral RNA copies per mL of sample (n = 4) (Fig. 3). Viremia was also observed on days 8, 9 and 12 p.i. in some animals, but no significant viral RNA was detected after day 12 p.i. The viremia measured in the serum of the infected animals varied depending on the animal (Table 3). Throughout the time viremia was detected, all animals exhibited inappetence and lethargy. The results indicated that ZIKV infection was established in all four marmosets.

**Table 3:** Non-human primates viremia after ZIKV infection.

Days	Animal	RNA copies/mL
−1	NHP 1	Negative
	NHP 2	Negative
	NHP 3	Negative
	NHP 4	Negative
0	NHP 1	841,90
	NHP 2	Negative
	NHP 3	Negative
	NHP 4	Negative
2	NHP 1	200,01
	NHP 2	Negative
	NHP 3	1075,95
	NHP 4	204,24
3	NHP 1	1149,61
	NHP 2	213,74
	NHP 3	237,92
	NHP 4	2191,26
4	NHP 1	2848,23
	NHP 2	625,86
	NHP 3	786,27
	NHP 4	3295,71
6	NHP 1	19958,28
	NHP 2	1761,87
	NHP 3	30199,58
	NHP 4	4156,29
9	NHP 1	129,26
	NHP 2	216,64
	NHP 3	Negative
	NHP 4	Negative
12	NHP 1	579,66
	NHP 2	229,28
	NHP 3	Negative
	NHP 4	9840,11
15	NHP 1	2208,29
	NHP 2	Negative
	NHP 3	Negative
	NHP 4	Negative
19	NHP 1	Negative
	NHP 2	346,82
	NHP 3	Negative
	NHP 4	Negative

To evaluate whether immunity elicited by the primary ZIKV infection would protect the marmosets against subsequent ZIKV infection, we challenged marmosets 1 and 2 (previously infected with 10^5^ and 10^6^ PFU/mL) subcutaneously with 5 × 10^5^ PFU/mL of ZIKV strain HS-2015-BA-01, 8 months after the first infection. Viremia was accessed before infection (day −1) and on days 3, 4, 5, 6 and 9 p.i. Marmosets did not develop viremia suggesting sterilizing immunity (Supplementary Fig. S2). We revisited the NHPs and collected blood samples 10 months after the second infection to perform neutralization assays. We observed that the neutralizing antibodies titers (PRNT_50_) were 1:320 and 1:160 respectively.

### Haematological parameters in infected *Callithrix penicilata*

**Figure 4:**
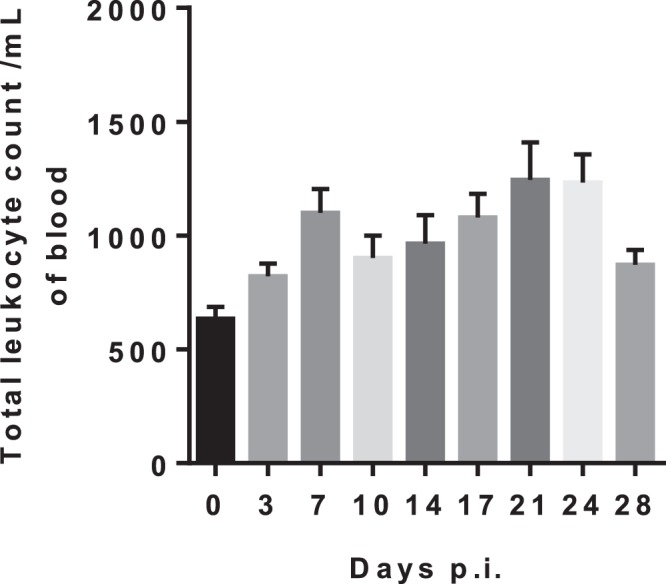
ZIKV infection in *Callithrix penicilata* alters total leukocyte counts. The animals were followed for 28 days. The data represents the results obtained of a pooled sample from four marmosets prior to infection (day 0) and in different days post infection (dpi). The infection altered total leukocyte count in blood inducing leukocytosis 7 dpi and 21 dpi in a two-wave fashion. Results are presented as counts/mm^3^ per ml of blood.

**Figure 5:**
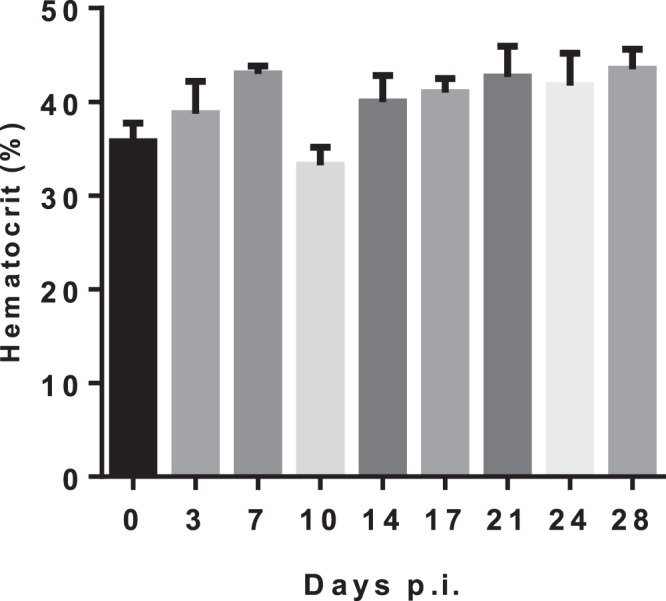
ZIKV infection in *Callithrix penicilata* alters hematocrit levels. The animals were followed for 28 days. The data represents the results obtained of a pooled sample from four marmosets prior to infection (day 0) and on different days post infection (dpi). No differences were observed in the hematocrit levels during the course of the infection. Results are presented as hematocrit percentage.

ZIKV infection in *Callithrix penicilata* induced leukocytosis in the blood of infected animals on days 7 and 21 p.i. in a two-wave fashion (Fig. 4). No differences were observed in the hematocrit during the course of infection (Fig. 5).

### IgG titers in infected *Callithrix penicilata*

**Figure 6:**
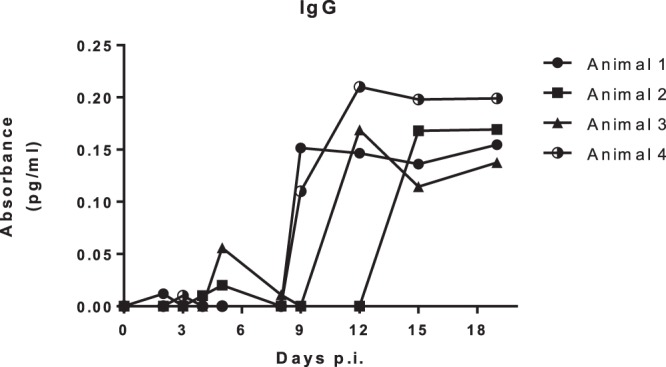
IgG titers in *Callithrix penicilata* after ZIKV infection. To evaluate the adaptive immune response after ZIKV infection, plasma samples were evaluated on days 0 (prior to infection) and 2,3,4,5,8,9,12,15,19 and 60 d.p.i. by an indirect ELISA. The IgG titers peaked from day 9 to 12 p.i. in the serum of marmosets 1,2 and 3 and on day 15 p.i. in the serum of marmoset 2 when compared to the other evaluated days. IgG titers were detectable until day 19 p.i. in all marmosets.

The adaptive immune response after ZIKV infection was evaluated by IgG titers in the serum of the ZIKV-infected marmosets. The IgG titers in the serum of marmosets 1, 3 and 4, peaked from day 9 to day 12 p.i., whether the IgG titers of marmoset 2 peaked on day 15 p.i. IgG titers were detectable until day 19 p.i. in all marmosets (Fig. 6). IgG titers on day 60 p.i. resembled the titers observed on day 19 p.i. (data not shown).

## Discussion

ZIKV was introduced in Brazil sometime in 2013^17–20^ and reached epidemic levels by mid-2015 and soon after spread throughout the Americas with unexpected clinical presentations, such as congenital Zika syndrome (CZS) and Guillain-Barré Syndrome (GBS)^21^.

In Africa, OWPs are involved in the enzootic transmission of ZIKV [reviewed in^4^]. On the other hand, it is not known whether neotropical NHPs could act as natural reservoirs and amplification hosts contributing to the establishment and maintenance of a sylvatic cycle after the recent introduction of ZIKV in the Americas. *Callithrix* and *Sapajus* species have distinct geographic distributions along the different Brazilian biomes and importantly this species’ distribution range extends well within several urban settings^22,23^. Additionally, it was recently demonstrated that three other neotropical NHP species can support ZIKV infection and thus can serve as reservoir and amplification hosts into a sylvatic transmission cycle^11,13,16^.

In a close temporal gap (between 2016 and 2017), Brazil experienced both DENV and ZIKV outbreaks and the largest sylvatic yellow fever (sYF) outbreak in over 50 years, which spread throughout the Southeast region^24,25^. During the recent YFV outbreak, we received NHP carcasses from São Paulo and Minas Gerais States, and decided to investigate the natural infection of NHP by ZIKV. All animals were tested for YFV and all negative NHPs were tested for ZIKV. ZIKV-specific RT-qPCR amplification products were obtained from 32 out of 82 NHP carcasses. All ZIKV positive animals were negative for YFV in RT-qPCR and RT-nested-PCR and a subset that was tested by immunohistochemistry to YFV exposure, were also negative. Our results demonstrated that free-living marmosets and capuchin monkeys may be naturally infected with ZIKV in urban areas of Southeast Brazil (São Paulo and Minas Gerais States), regions of intense arboviral circulation^26–32^. A previous study demonstrated ZIKV RNA detection in sera and/or oral swabs (not further specified in prior studies) from free-living common marmosets (n = 4) and capuchin monkeys (n = 3), in Northeast Brazil^8^. Thus, our results strengthen the hypothesis of the participation of these animals in the transmission/circulation of the ZIKV in urban areas.

In order to confirm the results and to better characterize the ZIKV strains circulating, we attempted to sequence the genome of ZIKV detected in NHPs. Although initial RNA tissue samples from the animals studied were degraded, the enrichment protocol used was able to capture specific ZIKV sequences from four marmosets samples. This library preparation protocol is time consuming, and it proved to be useful for sequencing ZIKV from complex samples, including degraded tissues. Sequence analyses confirmed the detection of ZIKV, belonging to the newly emerged American lineage in marmosets in São José do Rio Preto.

The molecular investigation of ZIKV in different NHP organs suggested a widespread viral distribution. We detected the highest ZIKV loads, indirectly measured by the Cts, in the kidneys, brain and spleen, which is in accordance with previous observations in rhesus monkeys and common marmosets experimentally infected with ZIKV^13,16,33,34^. Not all organs showed the presence of ZIKV RNA (Table 1). We believe that this finding may be attributed to the quality of the tissue sent for analysis, since we received carcasses in different stages of decomposition and also due to the sensitivity of the test used, as observed previously^15^.

ZIKV was detected in different organs of naturally infected NHPs. It is known that there are no pathognomonic lesions associated with ZIKV infection, but we looked for histopathological features in the organs of ZIKV naturally infected NHPs. The microscopic lesions that were common included: chronic interstitial nephritis; various patterns of pneumonia; vacuolar hepatopathy and varying degrees of hemodynamic disturbances (congestion, edema and hemorrhage) in multiple organs, predominately in the lung. The pathologic significance of these microscopic lesions is unknown; they were interpreted as nonspecific since they commonly occur in ZIKV-negative animals and we believe they lack diagnostic usefulness. Li *et al*.^34^, detected lesions in the CNS and visceral organs of rhesus monkeys after experimental subcutaneous ZIKV inoculation. In the CNS, they observed perivascular cuffing in cerebrum and brainstem and inflammatory infiltrates in the liver and spleen. In the present study, only few NHPs presented minimal inflammation in the central nervous system and no developmental anomalies were evident. Differences between experimental and natural infections may be related to the primate species (OWM versus Neotropical); the natural disease course, may be affected by other debilitating conditions, such as parasitic infestations and trauma, which were observed in some NHPs investigated previously. Further studies are needed to delineate the pathologic signature of ZIKV infection in neotropical primates.

Since we observed ZIKV natural infection in NHPs, we hypothesized the Brazilian common marmoset would sustain ZIKV infection and if they would be a suitable and reliable sentinel and experimental model of ZIKV infection. It is known that ZIKV infection of rhesus and cynomolgus monkeys recapitulates many key clinical findings, including rapid clearance of acute viremia, early invasion of the CNS, and prolonged viral shedding^33,35–39^. In accordance, the common marmoset is regarded as a feasible experimental model for the study of arboviral infections such as DENV^40^ because they can be easily kept in captivity owing to their the small size and the lower maintenance costs^41^. In the present study, we were able to successfully infect four marmosets with a low passage clinical isolate of ZIKV (HS-2015-BA-01) from a viremic patient with symptomatic ZIKV infection in Bahia State/Brazil. The four common marmosets infected with ZIKV had a transient viremia lasting up to 5 days post infection and the IgG level started appearing on day 9 and plateaued on day 19 on forwards, which further corroborates previous observations^11,38,42^. The animals also developed long term neutralizing antibodies as measured by PRNTs months after the infection. The viral load in serum after infection was approximately 10^3^ relative RNA copies on day 5p.i. Similar to what is observed in our model, the viremic phase of ZIKV was previously reported in studies conducted with OWPs and NHPs where the peak of viremia occurred between 2–5 d.p.i.^11,34^, similar to that observed in humans^43^. No neurological abnormalities were observed in the infected animals. Hematological analysis revealed a mild leukocytosis in all animals, but no alteration in hematocrit. A potential explanation for this hematological alteration would be related to, in parts, to host immunological factors and it has been considered as a disease indicator. As demonstrated by Omatsu *et al*., (2012), NHPs infected with DENV presented humoral immune response to the infection although the authors suggested that the clinical parameters alterations were due to the individual features among each animal^44^. Given the experimental design, no histopathological or immunohistochemical examinations were conducted on experimentally infected NHPs, so no further conclusions could be drawn.

This study aimed to fill in a gap of knowledge on natural ZIKV infection dynamics by focusing on the potential role that free-living NHPs may play as amplification hosts in a hyper-endemic region for arboviruses (as CHIKV, DENV and ZIKV). We have demonstrated the natural infection in marmosets and capuchin monkeys and experimental infection of marmosets with ZIKV. All free-living NHPs positive for ZIKV (and YFV negative) analyzed here, were collected as carcasses and based on the histopatological findings ZIKV detection did not appear to be correlated with the cause of death. Most of the animals were found death in urban/periurban areas with evidence of trauma. Due to the sYF outbreak that was occurring at the time the animals were collected, and due to the fear and misinformation of the population about the YFV transmission, local and sanitary authorities reported that these animals showed signs of poisoning and physical aggression^45,46^. From this observation alone, we cannot conclude that ZIKV infection was the cause of death, however, based on our experimental ZIKV infection study, where animals showed signs of lethargy and decreased motility, it is possible that these may have contributed to their demise as they became vulnerable to external threats mentioned earlier.

Recently, it has been demonstrated that NHP (from urban/peri-urban areas, free-ranging and captive animals) from Northeastern and Central-western Brazil, presented ZIKV-specific (and other arboviral) neutralizing antibodies. Although, the samples presented low titers of antibodies, it suggested exposure to ZIKV and thus a possible involvement in the establishment of ZIKV sylvatic cycle^47,48^. However, caution should be exercised, since flavivirus serology is uninformative in settings where subjects have been exposed to several heterologous flavivirus infections, which reiterates the notion that conclusive evidence requires the detection and/or isolation of ZIKV as well as ability to infect and disseminate in biting primatophilic vectors. Thus, we conducted an experimental ZIKV infection where four common marmosets were successfully infected with ZIKV and developed viremia and antibodies. Our results are in agreement with the studies cited above and reaffirm that ZIKV infection results in asymptomatic or subclinical manifestations.

After geoprocessing analyses, we observed a cluster of free-living ZIKV-positive marmosets and *Ae*. *aegypti*, in São José do Rio Preto (Fig. 1D). The neighborhood where the marmosets and mosquitoes were collected is surrounded by an area of vegetation which allows the NHPs and vectors survival (Fig. 1C). Together, the city is an area known to have high densities of *Ae*. *aegypti* and *Culex* spp. mosquitoes and the circulation of several flaviviruses^30,31,49,50^. The detection of ZIKV (American lineage), in free-living NHPs in different locations of Southeast Brazil (São Paulo and Minas Gerais States), supplemented by the experimental observations in marmosets strongly suggest a possible role of NHPs in the ZIKV transmission cycle and maintenance in urban areas and alert for a possible establishment of a ZIKV sylvatic cycle in Brazil. It is important to note that due to anthropogenic interference, marmosets and capuchin monkeys have been widely introduced into urban regions, especially in southern and southeastern metropoleis, where nearly half of the total population of Brazil resides. Additional studies to analyze transmission chains, including the investigation of ZIKV infection in sylvatic mosquitoes would help to better understand ZIKV dynamics in urban and sylvatic environments, in Brazil.

## Methods

### Study Context

In early 2017, Southeast Brazil faced a sylvatic yellow fever (sYF) outbreak. We received carcasses of free-living NHP collected in a neighborhood (Vila Toninho) of São José do Rio Preto (São Paulo state), where our research group conducts a prospective dengue cohort study in the population of the neighborhood. All the animals were found dead and the first suspicion was that YFV might have been the cause of death. Some of the animals had signals of trauma caused physical aggression. Together with our ongoing population cohort study, mosquitoes are regularly collected and Breteau index (BI) is performed to monitor the incidence of dengue in the neighborhood. RNA originated from the tissue samples (mosquitoes and NHP), was analyzed for the presence of YFV, ZIKV and DENV (only mosquitoes) genomes. After the positive results obtained from the specimens collected in Vila Toninho, we decided to investigate others urban/periurban free-living NHP carcasses collected in São José do Rio Preto, state of Sao Paulo and other cities of Minas Gerais state, due to the presence of a urban population of NHPs and the history of arboviruses circulation. They were submitted to the same analysis regarding the presence of YFV and ZIKV genomes. Based on previous evidence of experimental and natural infection of neotropical NHPs^8,11^, we performed experimental infections in captive marmosets to verify whether these NHPs could sustain ZIKV infection and viremia.

### Investigation of natural infection of NHP with ZIKV

#### Sample collection

NHP carcasses: A total of 82 carcasses belonging to free-living NHP (81 marmosets and one capuchin) were received from different urban and periurban areas of São José do Rio Preto (SP), from January to March 2017, and from cities of Minas Gerais State, from January to June 2017. These NHP were subjected to standardized necropsies, performed by local authorities on health services under the yellow fever national surveillance program (YFNSP). Life history and epidemiological data were recorded for each specimen in the National Injury Information Notification System (SINAN). Tissue samples including brain, gonads, heart, kidneys, liver, lungs and spleen were collected and submitted to the Laboratory of Virology (LPV), in São José do Rio Preto (SP), to Laboratório de Vírus/UFMG, in Belo Horizonte (MG), and to Adolfo Lutz Institute (IAL-SP), which take part on active surveillance of arboviruses.

*Aedes* mosquitoes: *Ae*. *aegypti* and *Ae*. *albopictus* mosquitoes were collected in the first trimester of 2017 (always in the last week of each month), in the Vila Toninho neighborhood, in an area close to the regions where some NHP carcasses were found. The mosquitoes were collected in BG-Mosquitito^TM^ traps, installed in the peridomestic area of selected residences, remaining in each residence for 24 hours. The traps were in covered and protected places, next to pots or foliage, hanging perpendicular to the ground, to approximately 1.5 meters of height. The mosquitoes were identified by taxonomic keys^51,52^ and the specimens were separated into pools with a maximum of 10 specimens per tube based on the site and collection date, and stored at −80 °C.

YFV and ZIKV molecular analysis:Samples of serum, brain, gonads, heart, kidneys, liver, lungs and spleen were subjected to molecular analysis to viral detection. Viral RNA was extracted with TRIzol® reagent (Thermo Fisher Scientific, Massachusetts, USA) and/or QIAamp Viral RNA Mini Kit (Qiagen, Hilden, Germany), according to the manufacturer’s instructions. A 50 mg of fresh or frozen tissues were initially macerated with 200 µl of TRIzol® reagent using autoclaved polypropylene pistils. Afterwards, 800 µL of TRIzol® reagent was added to homogenize the macerated samples and processed as the manufacturer’s instructions. To prevent contamination, recommendations were followed as described by Ausubel *et al*.^53^. The tissue manipulation was performed in a laminar flow hood. Detection of ZIKV RNA employed a TaqMan® reverse transcriptase quantitative polymerase chain reaction (RT-qPCR), as previously described with primers targeting the envelope gene ZIKV 1086 (CGCTGCCCAACACAAG); ZIKV 1162c (CCACTAACGTTCTTTTGCAGACAT) and probe ZIKV 1107-FAM (AGCCTACCTTGACAAGCAGTCAGACACTCAA)^43^, using the GoTaq® Probe 1-Step RT-qPCR System (Promega, Madison, USA) and/or QuantiNova Probe RT-PCR Kit (Qiagen, Hilden, Germany) according to the manufacturers’ instructions. The qPCRs using the Thermocycler QuantStudio 3 Real-Time PCR System (Thermo Fisher Scientific, Massachusetts, USA), ABI Step one, and/or ABI 7500 System (Applied Biosystems, Foster City, CA). Results were interpreted as cycle threshold (Ct)≤ 38.5 = positive; Ct > 38.5 or undetermined = negative)^43^. The YFV detection followed previous described protocols based on one-step TaqMan® RT-qPCR and RT-nested-PCR methodologies^54,55^. In the RT-nested-PCR, the thermal cycling was performed following previous report^55^. Precautions to avoid contamination were followed, and positive and negative controls were used in all reactions. The viral strains used as positive controls were the ZIKV^BR^ (Bioscience Institute, USP, Brazil) and the YFV 17DD vaccine (Biomanguinhos, FIOCRUZ, Brazil).

Viral RNA was extracted from mosquito pools with TRIzol reagent as described by Machado *et al*., 2012^56^. Briefly, 50 µL of 1X PBS were used for the initial maceration with autoclaved polypropylene pistils, followed by the addition of 850 µL of 1X PBS to homogenize the sample and centrifuged for 4 minutes at 2,300 g at 4 °C. Following centrifugation 400 µL of the clarified solution was placed in a clean 2.0 mL RNAse- and DNAse-free tube and along with the remainder sample stored at 80 °C. To the 400 µL sample, 1.2 mL of Trizol (Life Technologies - USA) was added and let it incubate at room temperature (RT) for 30 min, followed by the addition of 200 µL of chloroform. Tubes were gently mixed and let incubate at room temperature for 3 minutes, followed by centrifugation at 9,300 g for 15 minutes at 4 °C. The clear upper aqueous layer containing total RNA was transferred to a new tube and 500 µL of 2-propanol were added followed by gentle mix and incubation for 30 minutes at RT and centrifugation at 9300 g for 15 minutes at 4 °C. At the end of centrifugation the supernatant was completely removed and the RNA pellet was allowed to air-dry at RT followed by the addition and re-suspension in 30 µL of RNAse-free water. To identify the presence of the DENV1–4, YFV and ZIKV genomes, we used the same methodologies described for clinical samples as described above^43,55^.

Nucleotide Sequencing and Phylogenetic Analysis: DNA Libraries were prepared for deep sequencing with a ZIKV enrichment kit containing specific probes for Zika virus genome sequencing. The kit is still in development and standardization by Illumina® (San Diego, CA, USA), and the sequencing was performed with technical support from the Illumina team.

First, RNA integrity was evaluated with the Agilent RNA 6000 Nano kit in the 2100 Bioanalyzer Instrument (Agilent, Santa Clara, CA, USA), according to the manufacturer’s instructions. However, as the marmosets evaluated were found dead, samples were considered degraded and, therefore, not suitable for library preparation, according to the manufacturer’s instructions. On the other hand, those were the only samples available and we decided to continue with the library preparation protocol.

Second, the same concentration of RNA from different tissues from four free-living marmosets was pooled in order to increase the chance of ZIKV identification. Accordingly, spleen, kidney and lung from PR 17/05, spleen, lung, and blood clot from PR 17/19, liver, kidney, and lung from PR17/17, and brain and blood clot from PR 17/18 were pooled. Then, libraries were prepared with the Illumina®TruSeq® RNA Access Library Prep kit (Illumina, San Diego, CA, USA), with ZIKV-specific probes, according to the manufacturer’s instructions, skipping the first step that cleaves the RNA molecules. Libraries were validated using both the Agilent High Sensitivity DNA kit (Agilent) and a qPCR quantification system (Kapa, Kapa Biosystems, Wilmington, MA, USA). Sequencing run was performed with the MiSeq System using 2 nM denatured libraries and the respective reagent kit (1 × 75 cycles) (Illumina), according to the manufacturer’s instructions. A Zika virus strain isolated from a patient sample was used as positive control (GenBank accession number KY441403.1).

We combined the reads from all four samples to produce a consensus reference sequence and separately mapped the reads from each sample back to this reference. There was no evidence of any differences from the consensus in any of the individual samples. Total reads in each sample were 2.38, 4.48, 1.8 and 5.58 million, and mapped viral reads were 523, 1606, 392 and 1462 for samples Zika_17_05, Zika_17_17, Zika_17_18 and Zika_17_19, respectively. Average depths of coverage per base over all the bases were 3.7, 11.2, 2.7 and 10.2, and the number of reference bases with no read coverage were 1019, 260, 2310 and 275 for the four samples.

The evolutionary reconstruction of the ZIKV sequences was inferred by using the Maximum Likelihood method based on the General Time Reversible model with Gamma distribution^57^ on a dataset of 64 ZIKV open reading frames (ORFs). The reliability of branching patterns was tested through 1000 bootstrap sampling. In parallel, a phylogenetic tree using the same alignment was also generated using Bayesian analysis (Supplementary material).

Histopathological and immunohistochemical analysis: For histopathological analysis, tissue from 16 NHP (15 marmosets and one capuchin from São José do Rio Preto) including brain, gonads, heart, kidneys, liver, lungs and spleen were fixed in 10% neutral buffered formalin, processed and stained with hematoxylin and eosin for light microscopic examination.

For YFV immunohistochemical (IHC) analysis, silanized slides with liver tissue sections were subjected to wet antigen retrieval in a pressure cooker (Tris-EDTA solution 100 mM, pH 9.0). Endogenous peroxidase was blocked with 6% hydrogen peroxide for 40 min. An *in house* primary polyclonal anti-YF antibody (dilution 1:40,000; derived from hyperimmune mouse serum) was used, followed by overnight incubation (18 h) at 4 °C. Signal amplification was achieved with HiDef Detection^™^ HRP Polymer System (Cell Marque/Sigma-Aldrich, California, USA) by 30 min at 37 °C, followed by visualization with diaminobenzidine (D-5637; Sigma, St. Louis, MO, USA). The slides were mounted with aqueous media. Known NWP and human positive and negative control tissues with omitted first-layer antibody were included.

### Geoprocessing

The information regarding the sites where nine NHP carcasses were found and where *Ae*. *aegypti* mosquitoes were sampled in Vila Toninho were geocoded with the TerraView program (INPE, São José dos Campos-SP, Brazil). The shapefiles were layered in the ArcGIS program (ESRI, Redlands, CA, USA), and spatial-temporal associations between the positive mosquitoes and primates were analyzed. In order to observe the results, thematic maps were constructed.

### Experimental Study

#### Virus stock

A low passage of ZIKV^BR^^58^ and ZIKV isolate (HS-2015-BA-01; 2015 - accession No. KX520666) from a viremic patient in Bahia State, Brazil were used. A virus stock was produced in the *Ae*. *albopictus* C6/36 cell line. Supernatant was harvested 96 hours post infection (hpi) and virus stocks were titered by plaque assay in Vero cells. The virus inoculum was removed, following the addition of an overlay media containing 1.5% w/v carboxymethylcellulose (Synth, São Paulo, Brazil) in 2% FBS v/v DMEM. Seven days post infection (dpi), plates were fixed, washed and stained with methylene blue (Synth, São Paulo, Brazil) 1% w/v. Results were expressed as PFU/mL.

#### Experimental infection in marmosets

Four male black-tufted marmosets (*Callithrix penicillata*) were obtained from a breeding colony at the ‘Biotério de Primatas não Humanos’(Belo Horizonte, MG, Brazil). The animals were of various ages, ranging from one to ten-years-old. Marmosets were divided in four groups NHP 1, NHP2, NHP3, NHP4 and were infected subcutaneously with 10^5^ (NHP 1 and NHP 3) or 10^6^ PFU (NHP 2 and NHP 4) of ZIKV (HS-2015-BA-01) diluted in 500 µL of saline 0.9%. The animals were housed individually in four different cages (31cm-long,76cm-high and62cm-wide) and observed daily for 28 days. They were kept under a natural light *regime* under specific pathogen-free conditions. To evaluate whether immunity elicited by a primary ZIKV infection, NHP1 and NHP 2 were re-infected subcutaneously with 5 10^5^ PFU of ZIKV (HS-2015-BA-01) eight months later after the first infection (Supplementary material).

#### Plaque Neutralization Test

The Vero E6 cell line was maintained in Eagle’s Minimum Essential Medium (MEM, Cultilab, Brazil) at 37 °C in a humidified atmosphere containing 5% CO_2_. Sera samples assayed to determine the specific neutralization antibody titers based on a modified protocol described previously^59^. Briefly, the PRNT was performed in 24-well plates with 80,000 Vero E6 cells/well, using a fixed ZIKV^BR^ virus inoculum (~800 PFU) against varying serum dilutions (1:20-1:640). The plates were overlaid with a semi-solid medium (MEM 1 × , 1% FBS, 1.5% carboxymethylcellulose) and incubated at 37 °C in 5% CO_2_ for 5 days. The cell monolayer was fixed with 10% formalin solution and stained with 1% crystal violet solution. The PRNT titers were scored as reciprocal of the highest dilution of serum that inhibited 50% of plaques (PRNT_50_). Samples scored as PRNT 50 < 20, were considered negative.

#### Biological specimens collection and analysis

Whole blood samples were collected from the right femoral vein in EDTA-containing tubes. Blood samples were collected in different days either prior (day 0) or after the infection (from 2 to 28 dpi) for hematological analysis such as total leukocyte count (TLC), hematocrit analysis and for viral quantification by RT-qPCR.

#### Viral quantification by RT-qPCR

Viral RNA was obtained using the RNeasy Kit (Qiagen) and the One-step qRT-PCR was performed using QuantiNova Probe RT-PCR Kit (Qiagen, Heiden, Germany). Primers/probe sets specific for ZIKV were designed as described^43^. The standard curve was created using ten-fold dilutions of an international World Health Organization (WHO) Zika virus standard ranging from 5 × 10^2^ to 5 × 10^6^ genome copies/mL designed as described^60,61^. Amplification was performed on an ABI 7500 System (Applied Biosystems, Foster City, CA) using the following procedure: 45 °C for 10 min, 95 °C for 5 min, and 45 cycles at 95 °C for 5 s and 60 °C for 30 s. Results were expressed as genome copies/mL.

#### Haematological parameters

For total leucocyte count, the whole blood was diluted at 1:40 in TURK (IMBRALAB®) solution and the total cell count quantified in an optical microscope using a Neubauer chamber. Also, to determine the hematocrit, a sample of whole blood was collected into heparinized capillary tubes (Perfecta, São Paulo, Brazil) and centrifuged for 10 min in a hematocrit centrifuge (Fanem, Sao Paulo, Brazil). Results are presented as counts/mm^3^ per mL of blood (cells) or percentage. The rest of the blood was centrifuged for 10 min at 400 × g to collect the plasma. Plasma samples were stored frozen at −70 °C for RT-qPCR analysis and at −20 °C for indirect enzyme-linked immunosorbent assay (iELISA)assay.

#### Indirect ELISA

The indirect (i)ELISA to measure the immunoglobulin (Ig)-G titers in sera from the infected marmosets was performed as described with modification^62^. Briefly, ZIKV isolate (HS-2015-BA-01) was UV-inactivated and diluted in 0.01 M carbonate buffer (pH 9.6) to a concentration of 5 × 10^5^ PFU per well and incubated overnight. The plate was washed three times and blocked with 1% nonfat dry milk for 2 h. Serum was diluted (1:100), plated in duplicate, and incubated for 3 h. After another wash, plates were incubated for 2 h with an anti-human IgG (Fc specific) −peroxidase antibody (A0170 Sigma) diluted to 1:2000. Ortho-phenylenediamine was used as a substrate, and the reaction was stopped with 1 M sulfuric acid. Absorbance was measured at 492 nm. Samples of ZIKV-infected marmosets were considered positive in the first dilution in which mock samples were negative, and results were expressed as optical densities (OD).

### Ethics

The black-tufted marmosets were provided by the Brazilian Institute of Environment and Renewable Natural Resources (IBAMA) and accommodated at ‘Biotério de Primatas não Humanos’ (city of Belo Horizonte, MG, Brazil). They were certified by the System of Authorization and Information on Biodiversity (SISBIO) under the protocol number: 3106.6995/2012-MG. The experimental protocol was submitted to the Committee on Animal Ethics (CEUA) of the UFMG, with permit protocol number: 98/2017. Studies with ZIKV were conducted under Biocontainment level 2 (BCL2) at the ‘Laboratório de Imunofarmacologia at the Instituto de Ciências Biológicas (ICB) from the UFMG. All methods were performed in accordance with the guidelines and regulations described by CONCEA (2015)^63^.

## Electronic supplementary material

Supplementary Information
