# Supplementary material for: Evidence of natural Zika virus infection in neotropical non-human primates in Brazil
Source: Sci Rep. 2018 Oct 30;8:16034. doi: 10.1038/s41598-018-34423-6 (PMC6207778; doi:10.1038/s41598-018-34423-6)
Supplement: Supplementary file 1 — Supplementary Information [file 41598_2018_34423_MOESM1_ESM.pdf]

Supplementary material

Full title: **Evidence of natural Zika virus infection in neotropical non-human primates in Brazil.**

Ana Carolina B. Terzian[1], Nathalia Zini[1], Lívia Sacchetto[2], Rebeca Froes Rocha[3], Maisa Carla Pereira Parra[1], Juliana Lemos Del Sarto[3], Ana Carolina Fialho Dias[3], Felipe Coutinho[3], Jéssica Rayra[3], Rafael Alves da Silva[1], Vivian Vasconcelos Costa[3], Natália Coelho Couto de Azevedo Fernandes[4], Rodrigo Réssio[4], Josué Díaz-Delgado[4], Juliana Guerra[4], Mariana S Cunha[4], José Luiz Catão-Dias[5], Cintia Bittar[6], Andréia Francesli Negri Reis[7], Izalco Nuremberg Penha dos Santos[7], Andréia Cristina Marascalchi Ferreira[7], Lilian Elisa Arão Antônio Cruz[7], Paula Rahal [6], Leila Ullmann[8], Camila Malossi[8], João Pessoa de Araújo Junior[8], Steven Widen[9] , Izabela Maurício de Rezende[2], Érica Mello[10], Carolina Colombelli Pacca[11], Erna Geessien Kroon[2], Giliane Trindade[2], Betânia Drumond[2], Francisco Chiaravalloti-Neto[12]; Nikos Vasilakis[13], Mauro M Teixeira[3], Maurício Lacerda Nogueira[1*].

Phylogenetic analysis using Bayesian approach.

The four ZIKV sequences obtained here were aligned with ZIKV genome sequences (as described in the Methods section), from Asian and African genotypes. Bayesian analysis were carried out using BEAST package v.1.8.2 (Drummond et al, 2002-2015) with Markov Chain Monte Carlo algorithms. Input files for BEAST were created with BEAUTi 1.8.2 (Drummond et al, 2002-15). Runs were performed using the Constant model, under the relaxed (uncorrelated lognormal) molecular clock. One hundred million chains were run and the first 10 million steps were discarded. Convergence of parameters was verified with Tracer v.1.5.0 (Rambaut and Drummond AJ, 2003-2009) and uncertainties were addressed as the 95%Bayesian credible intervals (BCI). The trees were sampled at each 10,000 steps and then summarized in a maximum clade credibility tree using TreeAnotator v.1.8.2 (Rambaut and Drummond AJ, 2002-2015). The final tree was visualized in FigTree v.1.4.3 (Rambaut, 2006-2016). After the analysis, the ZIKV sequences obtained from the 04 NHP clustered together (supported by posterior probability = 1), within the Americam Zika lineage (Suplementary Figure S1), apart from African lineage (data not shown). The results confirm the previous ones obtained by Maximum likelihood approach.

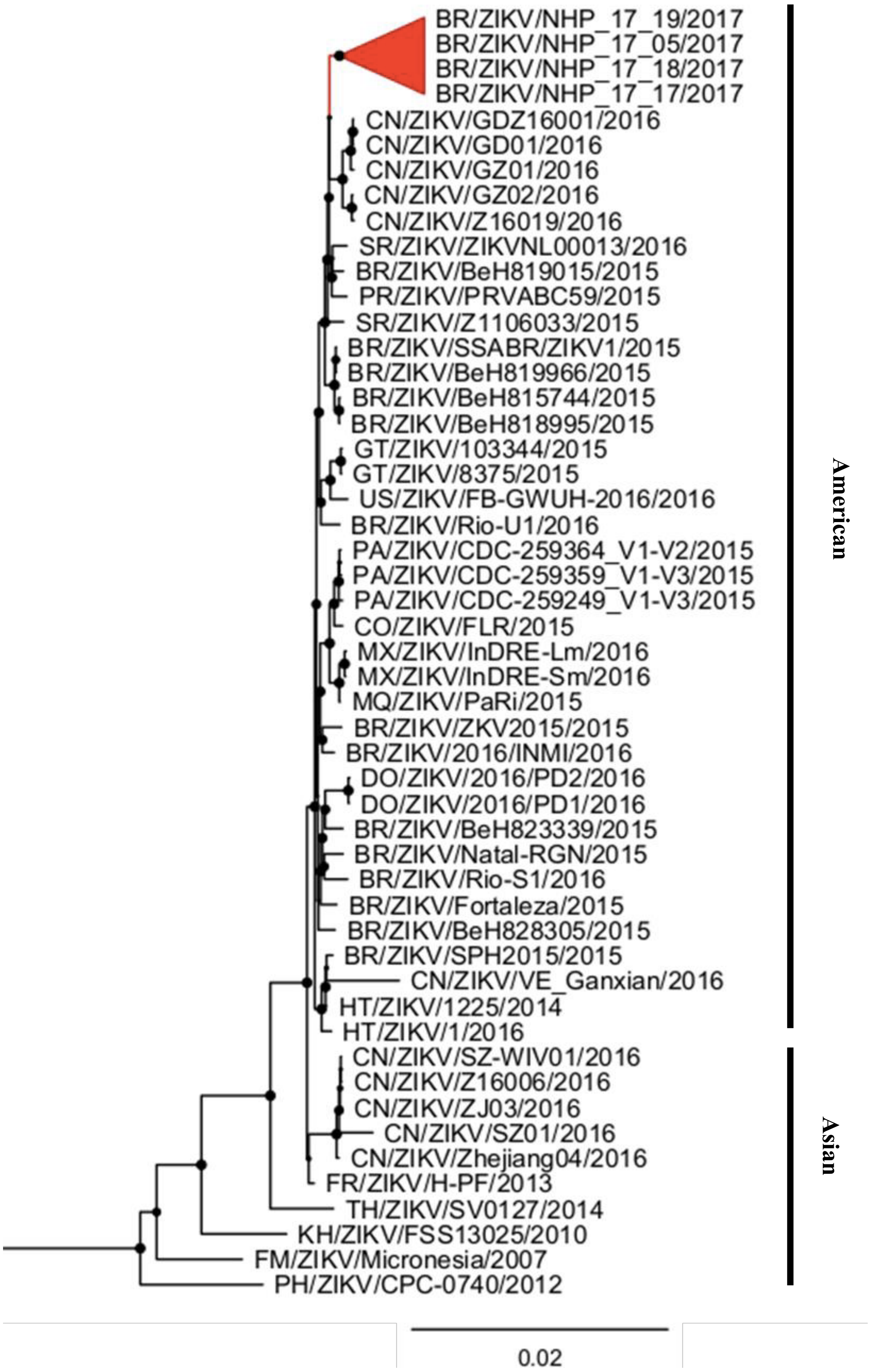
BR/ZIKV/NHP_17_19/2017
BR/ZIKV/NHP_17_05/2017
BR/ZIKV/NHP_17_18/2017
BR/ZIKV/NHP_17_17/2017
CN/ZIKV/GDZ16001/2016
CN/ZIKV/GD01/2016
CN/ZIKV/GZ01/2016
CN/ZIKV/GZ02/2016
CN/ZIKV/Z16019/2016
SR/ZIKV/ZIKVNL00013/2016
BR/ZIKV/BeH819015/2015
PR/ZIKV/PRVABC59/2015
SR/ZIKV/Z1106033/2015
BR/ZIKV/SSABR/ZIKV1/2015
BR/ZIKV/BeH819966/2015
BR/ZIKV/BeH815744/2015
BR/ZIKV/BeH818995/2015
GT/ZIKV/103344/2015
GT/ZIKV/8375/2015
US/ZIKV/FB-GWUH-2016/2016
BR/ZIKV/Rio-U1/2016
PA/ZIKV/CDC-259364_V1-V2/2015
PA/ZIKV/CDC-259359_V1-V3/2015
PA/ZIKV/CDC-259249_V1-V3/2015
CO/ZIKV/FLR/2015
MX/ZIKV/InDRE-Lm/2016
MX/ZIKV/InDRE-Sm/2016
MQ/ZIKV/PaRi/2015
BR/ZIKV/ZKV2015/2015
BR/ZIKV/2016/INMI/2016
DO/ZIKV/2016/PD2/2016
DO/ZIKV/2016/PD1/2016
BR/ZIKV/BeH823339/2015
BR/ZIKV/Natal-RGN/2015
BR/ZIKV/Rio-S1/2016
BR/ZIKV/Fortaleza/2015
BR/ZIKV/BeH828305/2015
BR/ZIKV/SPH2015/2015
CN/ZIKV/VE_Ganxian/2016
HT/ZIKV/1225/2014
HT/ZIKV/1/2016
CN/ZIKV/SZ-WIV01/2016
CN/ZIKV/Z16006/2016
CN/ZIKV/ZJ03/2016
CN/ZIKV/SZ01/2016
CN/ZIKV/Zhejiang04/2016
FR/ZIKV/H-PF/2013
TH/ZIKV/SV0127/2014
KH/ZIKV/FSS13025/2010
FM/ZIKV/Micronesia/2007
PH/ZIKV/CPC-0740/2012
American
Asian
0.02

Supplementary Fig. S1: Bayesian analysis of ZIKV from non-human primates. A total of 64 sequences (10269 bp) were aligned and phylogenetic history was inferred using Beast1.8.2. The subtree containing the Asian and American lineages is shown here. Sequences are indicated by their Genbank acession numbers. Dots (in scale) in the nodes represents the values of posterior probability. ZIKV sequences from NHP are shown in red. The sub-tree is drawn to scale (nucleotide/substitutions/year).

Experimental re-infection in marmosets.

To evaluate whether immunity elicited by a primary ZIKV infection, NHP1 and NHP 2 were re-infected subcutaneously with 5 10^5 PFU of ZIKV (HS-2015-BA-01), diluted in 500µL of saline 0.9%, eight months later after the first infection. The animals were housed individually in two different cages (31cm-long,76cm-high and62cm-wide) and observed daily. They were kept under a natural light *regime* under specific pathogen-free conditions. The experimental protocol was approved by the Committee on Animal Ethics (CEUA) of the UFMG, with permit protocol number:98/2017.

**Days**	**ZIKV RNA genome copies/ml**	
	**NHP 1**	**NHP 2**
**-1**	Negative	Negative
**0**	Negative	Negative
**3**	Negative	Negative
**4**	Negative	Negative
**5**	Negative	Negative
**6**	Negative	Negative
**9**	Negative	Negative

Supplementary Fig. S2: Viremia measurement in *Callithrix penicilata* after ZIKV re-infection. Samples were collected from day -1 until nine dpi. One-step qRT-PCR was used to measure semi quantitatively ZIKV RNA loads in the serum of NHP 1 collected at the indicated days p.i. and they are represented as viral RNA copies per mL of sample standard curve. The curve was obtained from a standard sample with known quantification after serial dilutions ($5x10^2$ to $5x10^6$ copies/mL) on the plasma of the non-infected marmosets. Values are expressed by RNA genome copies per mL for all the infected marmosets. for all the infected marmosets. p.i.: post infection. NHP: non-human primates. Day -1: day before the infection.

.

Supplementary references

Drummond AJ, Rambaut A, Suchard MA, Xie W. Bayesian Evolutionary Analysis Utility. Version v1.8.2, 2002-2015

Drummond AJ, Rambaut A, Suchard MA, Xie W. Bayesian Evolutionary Analysis Sampling Trees Version 1.8.2, 2002-15

Rambaut A, Drummond AJ (2003-2009) Tracer: MCMC Trace Analysis Tool Version v1.5.0. Available: http://beast.bio.ed.ac.uk/.

Rambaut A, Drummond AJ (2002-2015) TreeAnotator v1.8.2. Available: http://beast.bio.ed.ac.uk/.

Rambaut A (2006-2016) Tree Figure Drawing Tool Version 1.4.3. Available: http://tree.bio.ed.ac.uk/.
